# Restriction of HIV-1 Replication in Monocytes Is Abolished by Vpx of SIVsmmPBj

**DOI:** 10.1371/journal.pone.0007098

**Published:** 2009-09-21

**Authors:** Silke Schüle, Björn-Philipp Kloke, Julia K. Kaiser, Sabine Heidmeier, Sylvia Panitz, Nina Wolfrum, Klaus Cichutek, Matthias Schweizer

**Affiliations:** Division of Medical Biotechnology, Paul-Ehrlich-Institut, Langen, Germany; University of Minnesota, United States of America

## Abstract

**Background:**

Human primary monocytes are refractory to infection with the human immunodeficiency virus 1 (HIV-1) or transduction with HIV-1-derived vectors. In contrast, efficient single round transduction of monocytes is mediated by vectors derived from simian immunodeficiency virus of sooty mangabeys (SIVsmmPBj), depending on the presence of the viral accessory protein Vpx.

**Methods and Findings:**

Here we analyzed whether Vpx of SIVsmmPBj is sufficient for transduction of primary monocytes by HIV-1-derived vectors. To enable incorporation of PBj Vpx into HIV-1 vector particles, a HA-Vpr/Vpx fusion protein was generated. Supplementation of HIV-1 vector particles with this fusion protein was not sufficient to facilitate transduction of human monocytes. However, monocyte transduction with HIV-1-derived vectors was significantly enhanced after delivery of Vpx proteins by virus-like particles (VLPs) derived from SIVsmmPBj. Moreover, pre-incubation with Vpx-containing VLPs restored replication capacity of infectious HIV-1 in human monocytes. In monocytes of non-human primates, single-round transduction with HIV-1 vectors was enabled.

**Conclusion:**

Vpx enhances transduction of primary human and even non-human monocytes with HIV-1-derived vectors, only if delivered in the background of SIVsmmPBj-derived virus-like particles. Thus, for accurate Vpx function the presence of SIVsmmPBj capsid proteins might be required. Vpx is essential to overcome a block of early infection steps in primary monocytes.

## Introduction

Compared to more differentiated cells of the hematopoietic lineage such as macrophages or dendritic cells (DCs), human primary monocytes are relatively refractory to HIV-1 infection *in vitro* [1], [2]. Likewise, lentiviral vectors derived from HIV-1 are not able to mediate efficient monocyte transduction, whereas transduction of monocyte-derived macrophages was possible at low levels [1], [3]. The reason for this difference is not clearly understood. HIV-1 vectors pseudotyped with the envelope glycoprotein of vesicular stomatitis virus (VSV) retain this different transduction capacity for monocytes or macrophages [1], suggesting that the block in monocytes is independent of viral entry by attachment to CD4 or HIV-1 coreceptors. In some studies the block is described as occurring prior to reverse transcription [4], [5], while others suggest that in monocytes an early post entry block occurs shortly after reverse transcription [5], [6]. At least the viral cDNA synthesis and integration seem to be *per se* extremely inefficient in monocytes since differentiation-dependent cofactors of reverse transcription might be limited [4], [7]. After differentiation of monocytes into DCs, vectors that had already entered the cells were rescued, whereat activation might have induced accumulation of nuclear viral DNA [1]. Different activity of cellular restriction factors like APOBEC proteins [8]–[10] may contribute to the different susceptibility of monocytes and more differentiated cells to HIV-1.

Recently, we described efficient transduction of primary human monocytes using lentiviral vectors derived from the simian immunodeficiency virus strain SIVsmmPBj isolated from sooty mangabeys [3]. This capacity of SIVsmmPBj vectors depends on the viral protein Vpx, whereas all other accessory genes are dispensable [11]. A vector mutant designated PBj4xko-EGFP, which lacks expression of the accessory genes *vif*, *vpr*, *vpx* and *nef* was not capable of monocyte transduction. However, this capacity could be restored by supplementation of the PBj4xko-EGFP vector with Vpx [3], [11]. The Vpx protein is encoded by viruses of the HIV-2/SIVsmm/SIVmac lineage and it has been previously described that replication of these viruses or efficient transduction of macrophages or dendritic cells (DCs) with respective vectors strictly depends on the presence of this viral protein [3], [11]–[15]. A number of studies correlated the loss of nuclear localization of the Vpx protein with the inability of mutant viruses to infect non-dividing cells, thus arguing for its role in nuclear import, similar to Vpr of HIV-1 [12], [16]–[18]. To investigate whether Vpx is the only factor required for monocyte transduction with lentiviral vectors, we previously attempted to package Vpx of SIVsmmPBj into HIV-1 particles [11]. In order to achieve this, the p6 domain of the HIV-1 Gag protein was modified to allow binding and packaging of Vpx. However, the resulting Vpx-containing HIV-1 vector particles did not facilitate a more efficiently transduction of primary monocytes than the unmodified HIV-1 vector. The block occurred after reverse transcription of vector RNA and before translocation of the pre-integration complex (PIC) into the nucleus, similar to that shown for HIV-1 vectors or for PBj-derived vectors lacking Vpx [1], [3], [11], [19]. We concluded that Vpx might not be present in the HIV-1 PIC, when delivered with the modified HIV-1 particles, since Vpx may require specific binding to other virus-specific components like integrase, MA or RNA.

In the present study, two further strategies were conducted to confer Vpx properties to HIV-1-derived vectors. At first, a HA-Vpr/Vpx fusion protein was generated to link Vpx function to the HIV-1 counterpart Vpr in order to mediate packaging of Vpx via Vpr into HIV-1 vector particles and subsequent targeting to the PIC. Furthermore, we attempted to provide Vpx by preincubation of target cells with virus like particles (VLPs) containing Vpx. We analyzed whether Vpx functions enabled single-round infection and full replication of HIV-1 in human and simian monocytes. The results indicate that abolition of the HIV-1 restriction block in monocytes is enabled by Vpx but also requires SIVsmmPBj Gag or Pol functions.

## Results

### Packaging of SIVsmmPBj Vpx into HIV-1-derived particles using Vpr-Vpx fusion proteins

**Figure 1:**
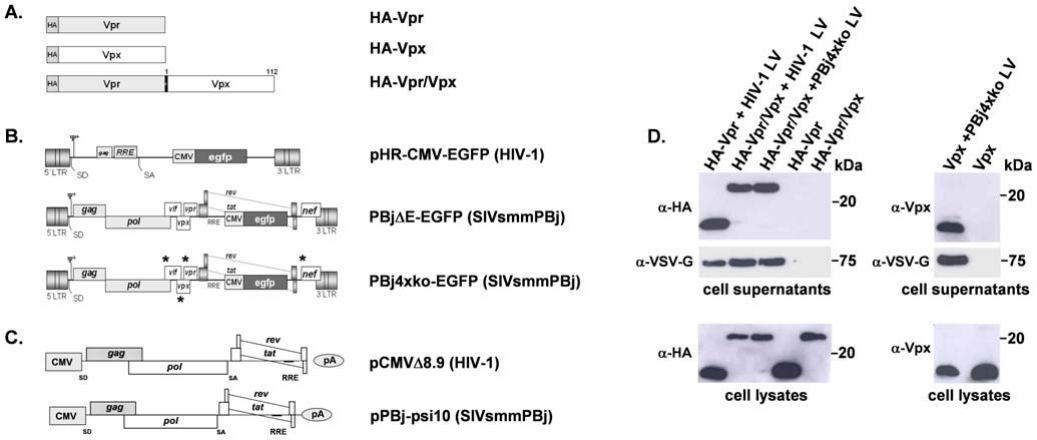
Schematic representation of expression constructs and packaging of Vpr/Vpx fusion proteins into vector particles was detected by Western-blotting. (A) The viral proteins HIV-1 Vpr, SIVsmmPBj1.9 Vpx and related fusion proteins encoded by the expression vector pcDNA3.1. For generation of the fusion protein, the full length Vpx was ligated to HIV-1 Vpr. HA, hemagglutinin tag. (B) HIV-1- or SIVsmmPBj- derived transfer vectors coding for EGFP. In the PBj4xko-EGFP vector, coding regions of all four accessory genes are knocked-out by insertion of stop-codons marked by an asterisk. (C) PBj- and HIV-1-derived packaging constructs coding for Gag-Pol and the regulatory proteins Tat and Rev. These constructs were also used for production of virus-like particles. For pseudotyping of vectors or virus-like particles, the vesicular stomatitis virus G protein (VSV-G, not shown) was used. (D) Western blots of supernatants or lysates of 293T packaging cells cotransfected with the respective vector constructs required for generation of VSV-G-pseudotyped lentiviral vectors (HIV-1 LV or PBj4xko LV), and one of the expression constructs encoding Vpr, Vpx or the Vpr/Vpx fusion protein as indicated on the top. As controls, supernatants and lysates of cells transfected only with one of the Vpr, Vpx or Vpr/Vpx constructs were analyzed. Cell supernatants were purified by filtering and sucrose cushion centrifugation. For labelling, antibodies directed against HA, Vpx, or the envelope protein VSV-G were used. Ψ, packaging signal; LTR, long terminal repeat; SD, splice donor; cPPT, central polypurine tract; RRE, rev responsive element; SA, splice acceptor; CMV, cytomegalovirus promoter; BGHpA, bovine growth hormone polyadenylation signal.

Previously, we have shown that the Vpx protein of SIVsmmPBj is not incorporated into unmodified HIV-1 vector particles [11]. A modified HIV-1 vector, which carried the Vpx interaction domain of the PBj Gag protein within the corresponding HIV p6 domain, was able to package Vpx. However, this Vpx-containing HIV-1 vector was not able to transduce primary human monocytes [11]. Monocyte transduction was achieved when the SIVsmmPBj-derived PBj4xko-EGFP was supplemented with the Vpx protein *in trans*. As the HIV-1 counterpart protein Vpr is packaged during virus budding into the particle and is part of the HIV-1 PIC [20], we aimed to enable co-localization of Vpx with HIV-1 Vpr in the target cell by generating a fusion protein consisting of HIV-1 Vpr and PBj Vpx. Schematic representations of the HA-tagged fusion construct as well as of the Vpr and Vpx proteins are shown in Fig. 1A.

For generation of HIV-1 vector particles, 293T cells were co-transfected with the transfer vector pHR-CMV-EGFP (Fig. 1B), the packaging construct pCMVΔR8.9 (Fig. 1C), the VSV-G expression plasmid pMD.G and an expression vector encoding for HA-Vpr, Vpx, or HA-Vpr/Vpx fusion protein. SIVsmmPBj vectors were generated likewise but using the vector PBj4xko-EGFP lacking all accessory genes (Fig. 1B). Vector particles were harvested from supernatant, purified by sucrose gradient centrifugation, and analyzed by Western-blotting for the presence of the respective Vpx or HA-Vpr proteins (Fig. 1D). The HA-Vpr/Vpx fusion protein was detected at similar proportions in HIV-1- and PBj4xko-EGFP-derived vector particles. Presence of HA-Vpr, Vpx or HA-Vpr/Vpx proteins in the supernatant fraction indicated successful packaging into vector particles, as in the absence of the Gag-Pol and the envelope constructs none of those proteins were detected. In lysates of packaging cells, comparable expression levels of the respective proteins were determined independently from the presence or absence of the packaging and envelope expression constructs.

To demonstrate integrity of the generated fusion protein, the subcellular localization was analyzed in HeLa cells. In the absence of other viral proteins, HA-tagged Vpr, Vpx and the Vpr/Vpx fusion protein are localized in the nucleus (supplementary Fig. S1), as previously described for similar constructs [12], [16], [21], [22] indicating that localization of the fusion protein as a prerequisite of Vpx function is retained.

### HIV-1 vector particles supplemented with the HA-Vpr/Vpx fusion protein are not able to transduce primary human monocytes

**Figure 2:**
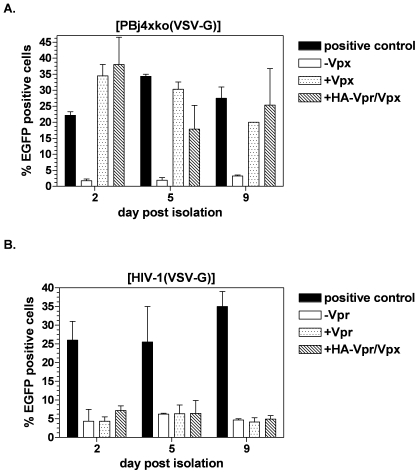
Packaging of Vpx into vector particles enhances transduction by SIVsmmPBj-derived but not HIV-1-derived vectors. Percentage of EGFP-expressing monocytes or monocyte-derived cells transduced at various days post isolation using VSV-G pseudotyped vector particles (MOI 10) supplemented with Vpx, Vpr, or the Vpr/Vpx fusion protein as indicated. The percentage of EGFP-expressing cells was determined by flow cytometry 6 days post transduction. (A) Transduction efficiency of PBj4xko-EGFP-derived vector particles. (B) Transduction efficiency of HIV-1-derived vector particles. Positive control: PBΔE-EGFP-derived vector containing all accessory genes. Means of three different donors are shown.

To investigate the capacity of HA-Vpr/Vpx containing HIV-1-derived vectors to transduce primary human monocytes, these cells were isolated from blood of healthy donors by negative depletion and analyzed by FACS for expression of the surface marker CD14. Routinely, the purity of the monocyte population was about 90% (data not shown). Purified cells were transduced at various days post isolation with an MOI of 10 using VSV-G pseudotyped SIVsmmPBj- or HIV-1-derived vector particles supplemented with the HA-Vpr/Vpx fusion protein, HA-Vpr or wild-type Vpx. At the first time point of transduction at day two after isolation, the cells are quiescent monocytes as confirmed by low DNA/RNA content [3].Three days after cultivation, the cells start to differentiate to macrophages, as indicated by increased DNA/RNA content, decreased expression of CD86 surface marker and change of morphology [3], and are designated as monocyte-derived cells. Independent of the day of transduction, application of PBj4xko-EGFP vector particles containing the HA-Vpr/Vpx fusion protein resulted in an approximately 10-fold higher transduction rate, compared to non-supplemented vector (Fig. 2A). The observed transduction efficiency was comparable to that of PBj4xko-EGFP vectors supplemented with wild-type Vpx, indicating that the generated HA-Vpr/Vpx fusion protein is entirely functional. In contrast, HIV-1 vectors containing the HA-Vpr/Vpx fusion protein were not capable of transducing primary human monocytes or monocyte-derived cells with higher efficiency than non-supplemented particles (Fig. 2B). These results indicate that further PBj-specific factors in addition to the Vpx protein might be required for efficient transduction of primary human monocytes.

### Vpx provided by SIVsmmPBj-derived virus-like particles enables HIV-1 vectors to transduce primary human monocytes

**Figure 3:**
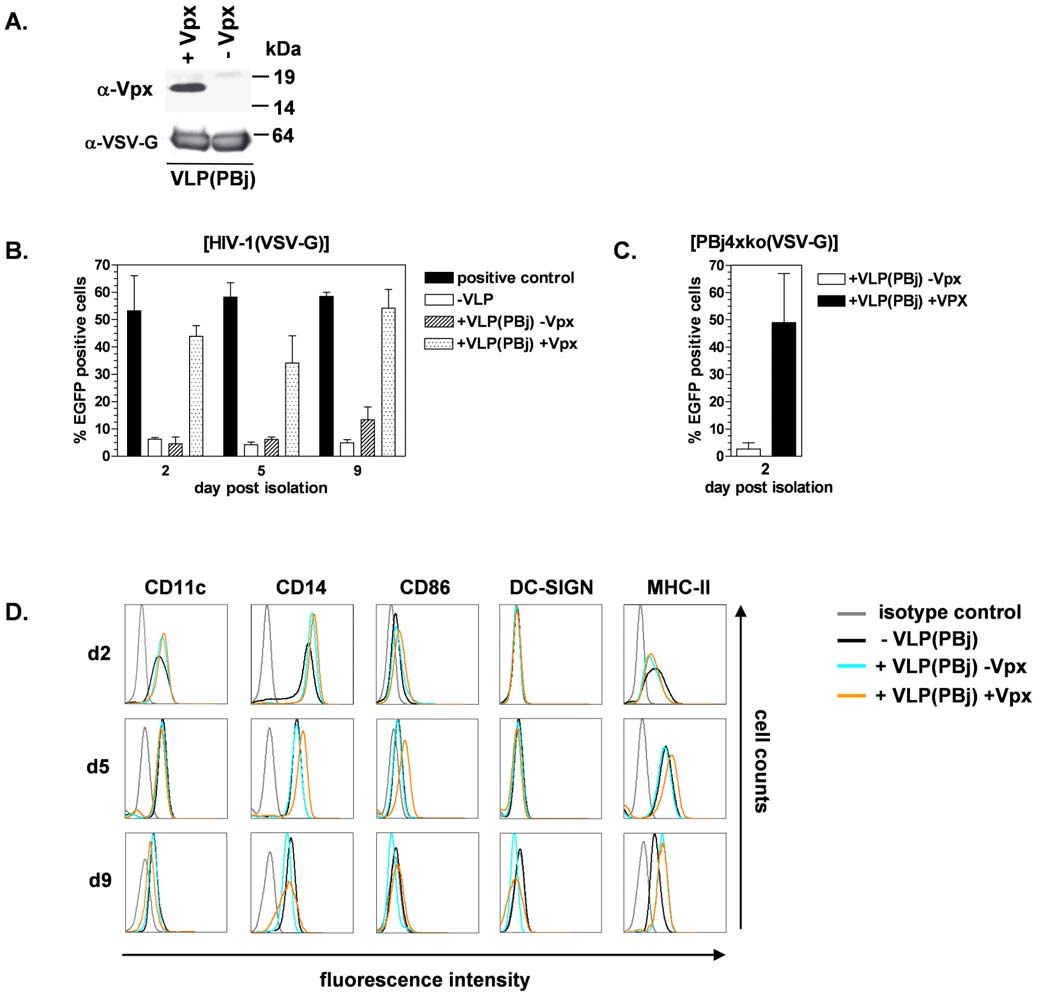
Vpx-containing SIVsmmPBj-derived VLPs enhance transduction with HIV-1 vectors. (A) Packaging of Vpx into PBj-derived VLPs was detected by Western-blotting. VLPs were generated by cotransfection of the PBj packaging vector pPBj-psi10 and the VSV-G and Vpx expression plasmids. Forty-eight hours post transfection, vector containing supernatant was collected from the 293T cells, filtered and purified twice by ultracentrifugation through a sucrose cushion. Purified VLPs were analyzed on Western blots using antibodies directed against Vpx or the envelope protein VSV-G. (B, C) Transduction of primary human monocytes or monocyte-derived cells 2 h after pre-incubation with Vpx-containing or non-containing PBj-derived VLPs (VLP(PBj)) at an MOIeq of 1 (determined by RT-Assay with standards of known infectivity) as indicated. Percentage of EGFP-expressing cells transduced at various days after isolation using (B) HIV-1 vectors or (C) PBj4xko-EGFP vectors. The percentage of transduced cells was determined by flow cytometry 6 days post transduction. The means of two different donors are shown. (D) Influence of Vpx on activation and differentiation of monocytes or monocyte-derived cells. At the indicated day after isolation, cells were incubated for 48 h with VLPs supplemented with Vpx (+VLPs +Vpx) or not (+VLPs −Vpx) at an MOIeq of 1, in comparison with untreated cells (−VLPs). The surface expression of the markers indicated was determined by FACS analysis. One representative donor out of three is shown.

Since packaging of Vpx into HIV-1 vector particles did not result in enhanced monocyte transduction, we attempted to provide Vpx by preincubation of monocytes with Vpx-containing virus-like particles. The VLPs were generated by transfection of 293T cells with the SIV-PBj-derived packaging construct pPBj-psi10 lacking all accessory genes (Fig. 1C), the VSV-G expression construct pMD.G and an expression construct encoding wild-type Vpx (Fig 1A). VLPs were purified from supernatant of transfected cells by sucrose gradient centrifugation, and the presence of Vpx was proven by Western blotting (Fig. 3A). To quantify VLPs, Reverse transcriptase activity was determined and compared to that of PBj vectors of known infectivity on HT1080 cells, assuming that RT activity is equal for infectious and non-infectious particles generated using the same vector genome. Accordingly, after preincubation of target cells with VLPs, the number of VLPs per cell was given as MOI-equivalents (MOIeq). Successive transduction experiments with monocytes using an HIV-1-derived encoding EGFP vector were carried out as described above, but monocytes or monocyte-derived cells were pre-incubated for two hours prior to transduction with VLPs at an MOIeq of 1 (Fig. 3B). Now, efficient transduction with HIV-1 vectors was observed with efficiencies slightly lower than those reached with the positive control vector PBjΔE-EGFP containing a functional Vpx. Pre-incubation with VLPs not supplemented with Vpx did not enhance transduction of monocytes or monocyte-derived cells with the HIV-1-derived vector. Transduction of monocytes with PBj4xko-EGFP vector particles was also achieved by pre-incubation of cells with Vpx-containing VLPs (Fig. 3C).

To determine a potential influence of Vpx on differentiation and/or activation of the cells, the surface expression of CD11c, CD14, CD86, DC-SIGN and MHCII was analyzed. Monocytes or monocyte-derived cells were incubated at various days after isolation for 48 h with VLPs carrying Vpx or not and analyzed by FACS (Fig. 3D). The slight overall decrease of CD11c, CD14 and CD86 suggested differentiation of monocytes to macrophages during the observation period [23], [24] which was confirmed by absence of the dendritic marker DC-SIGN. The antigen presenting molecule MHC-II and the co-stimulatory molecule CD86 were slightly increased on cells treated at d5 with Vpx-supplemented VLP, indicating a possible Vpx-dependent activation. However, beside these exceptions only marginal differences were detected between untreated and VLP-treated cells. In particular, d2 cells which are most relevant since they are clearly monocytes showed identical expression patterns after treatment with Vpx-carrying or empty VLPs, indicating that differentiation or activation of monocytes by Vpx plays only a minor role for transduction, if any.

**Figure 4:**
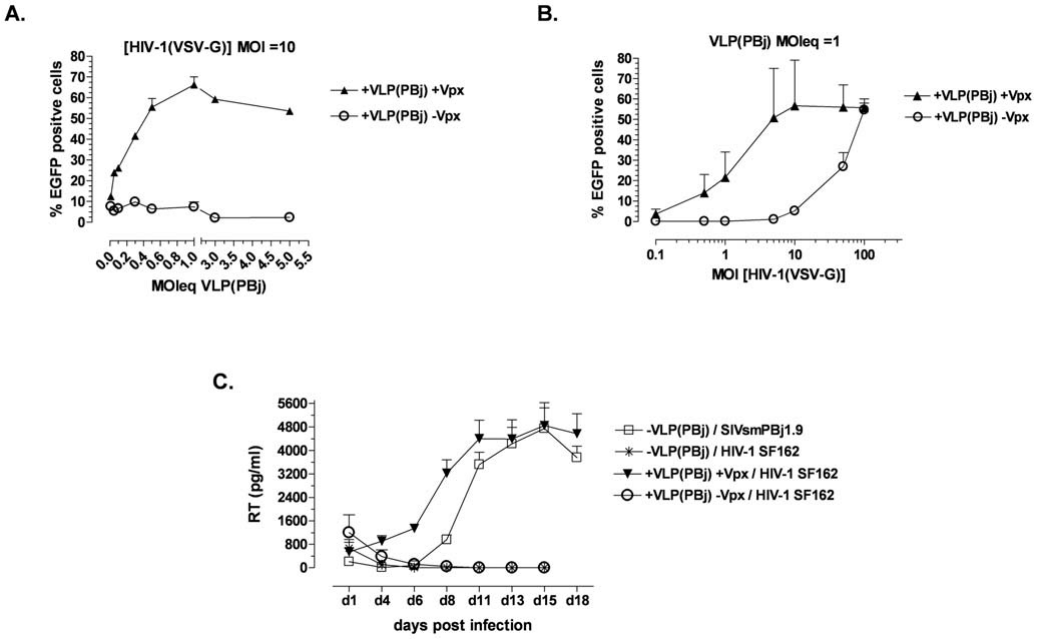
Enhancement of HIV-1 transduction of monocytes by PBj-derived VLPs is dose-dependent and enables replication of HIV-1 on human monocytes. Percentage of EGFP-expressing cells 6 days post transduction. (A) Monocytes (day 2 post isolation) were pre-incubated for 2 h with increasing amounts of PBj-derived VLPs (VLP(PBj)) as indicated, and transduced with HIV-1 vectors at an MOI of 10. (B) Monocytes were incubated with PBj-derived VLPs at an MOIeq of 1 (determined by RT-Assay with standards of known infectivity), followed by transduction with HIV-1 vectors at increasing MOI as indicated. The means of two different donors are shown. (C) On day 1 post isolation, monocytes were incubated for 2 h with Vpx-containing or non-containing VLPs at an MOIeq of 1 and subsequently infected with CCR5-tropic HIV-1 strain SF165 at an MOI of 0.5. SIVsmmPBj1.9 infection was performed without previous VLP incubation. Replication kinetics of HIV-1 or SIVsmmPBj after pre-incubation of monocytes with VLPs as indicated. Virus replication was assessed by quantifying RT activity in cell culture supernatants. Means of two different donors from one representative out of three experiments are shown.

To describe the effect in dependence of the amount of viral particles, either the amount of vectors or that of VLPs was varied. At first, increasing amounts of VLPs were used for pre-incubation of monocytes. An enhancing effect was already obtained at an MOIeq of 0.1. This effect increased dose-dependently up to an MOIeq of about 1 (Fig. 4A). Next, monocytes were preincubated with VLPs at an MOIeq of 1 and subsequently transduced using different amounts of HIV-1-derived vector particles (Fig. 4B). Transduction rates increased with increasing vector MOI. The highest transduction rate of about 50% was reached with an MOI of 10. In contrast, no transduction was detected after preincubation of monocytes with VLPs lacking Vpx, even at an MOI of HIV-1 vector particles of 10. Transduction efficiency increased only if higher amounts of HIV-1 vector particles were used. The maximum transduction efficiency of about 50% in the presence of Vpx was achieved with an MOI of 10, and in the absence of Vpx with an MOI of 100.

Since single round transduction with HIV-1-derived vectors is enabled by pre-delivery of SIV Vpx, we analyzed whether Vpx also facilitates full HIV-1 replication in these cells. Monocytes were pre-incubated at day 1 post isolation with PBj-derived VLPs either containing or lacking Vpx at an MOIeq of 1 for two hours and subsequently infected with the CCR5-tropic HIV-1 virus strain SF162 at an low MOI of 0.5. Replication was determined by measuring RT-activity in the supernatant of infected cells for 18 days. SIVsmmPBj1.9 wild-type virus which encodes Vpx and is known to efficiently replicate in primary macrophages [12], [15], [25] was used as a control. The replication capacity of the HIV-1 virus was completely rescued when monocytes were treated with Vpx containing VLPs, and was comparable to that of SIVsmmPBj1.9 on untreated cells. In contrast, HIV-1 did not replicate in untreated cells or after pre-incubation with VLPs not containing Vpx (Fig. 4C).

### Vpx provided by SIVsmmPBj-derived VLPs enables HIV-1 vectors to transduce primary simian monocytes

**Figure 5:**
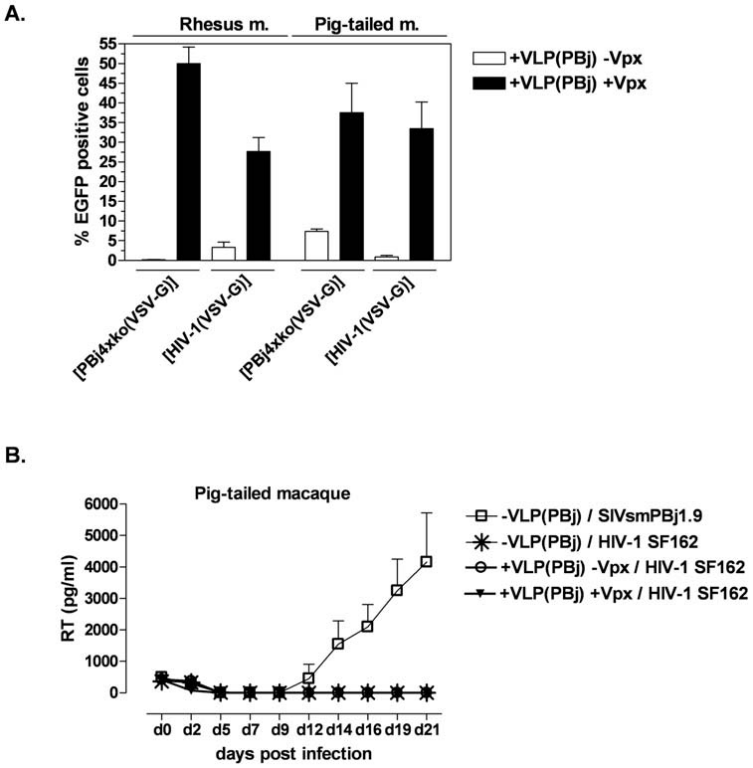
Vpx enables single-round but not full replication of HIV-1 in non-human primate monocytes. (A) Transduction of simian monocytes. Monocytes from Rhesus or Pig-tailed macaques were pre-incubated for 2 h with PBj-derived VLPs either containing or lacking Vpx at an MOIeq of 1, and subsequently transduced with HIV-1 or PBj4xko-EGFP vectors at an MOI of 7 as indicated. Means of three animals of each species are shown. (B) Replication kinetics of HIV-1 or SIVsmmPBj. Monocytes of Pig-tailed macaques were stimulated at day 1 post isolation for 8 h with TPA before incubation with VLPs as indicated at an MOIeq of 1, and subsequent infected an MOI of 0.5 with HIV-1 strain SF165 or SIVsmmPBj1.9. Virus replication was assessed by quantifying RT antigen in cell culture supernatants. Means of three different donors out of two independent experiments are shown.

With the exception of chimpanzees, non-human primates are naturally resistant to HIV-1 infection [26]–[29]. Restriction of HIV-1 infection in rhesus macaque cells *in vitro* has been attributed to a post-entry step, perhaps due to cellular restriction factors like TRIM5α or APOBEC proteins [30], whereas TRIM5α restriction has been described to be absent in Pig-tailed macaques [31]. Here, we investigated whether Vpx-containing VLPs can overcome these restrictions and enable transduction of simian monocytes with HIV-1-derived vectors. Monocytes of rhesus macaques (Macaca mulatta) or Pig-tailed macaques (Macaca nemestrina) were isolated, pre-incubated after two days with PBj-derived VLPs at an MOIeq of 1, and subsequently transduced with PBj4xko-EGFP- or HIV-1-derived vectors at an MOI of 8. Efficient transduction of non-human primate monocytes was achieved using both vector types after pre-incubation with Vpx-containing VLPs (Fig. 5A). The transduction efficiency was comparable to that of the SIVsmmPBj-derived PBj4xko-EGFP vector on Pig-tailed macaque monocytes, although the transduction capacity of rhesus macaque cells was slightly higher. In contrast, pre-incubation with VLPs lacking Vpx did not significantly increase HIV-1 transduction of simian monocytes. These results indicate that Vpx allows single-round infection of non-human primate cells with an HIV-1-derived vector.

Next we analyzed whether overcoming this first blocks of infection would allow HIV-1 replication of HIV-1 wild-type virus on non-human monocytes. Monocytes isolated from Pig-tailed macaques were stimulated for 8 h with TPA to enhance attachment and growth of cells. On day 1 post isolation, monocytes were pre-incubated with Vpx-supplemented or non-supplemented PBj VLPs at an MOIeq of 1 for two hours, and subsequently infected with the CCR5-tropic HIV-1 virus strain SF162 at an MOI of 0.5. Replicating SIVsmmPBj virus was used as control. Replication was determined by measuring RT activity levels in the supernatant of infected cells for 21 days. Results demonstrated that replication of the HIV-1 virus did not occur in monocyte-derived cells of Pig-tailed macaques (Fig. 5B), although single round infections of HIV-1 vectors was enabled in the presence of Vpx. Full replication was observed in control cells infected with SIVsmmPBj1.9 (Fig. 5B).

## Discussion

The data presented here emphasizes the essential role of Vpx during the infection of primary monocytes by lentiviral viruses. Primary human monocytes are relatively refractory to single round infection or full replication of HIV-1 [1], [3], albeit efficient infection of monocytes, DCs or macrophages can be achieved by using high amounts of input virus [23], [32], [33]. We described recently that transduction of monocytes with vectors derived from SIVsmmPBj [3], [11] is clearly enhanced in the presence of the viral protein Vpx, which is not encoded by the genome of HIV-1. To determine whether Vpx is the single factor within the SIVsmmPBj derived virus required for transduction of quiescent cells like monocytes, we decided to assign the functional activities of Vpx to HIV-1. To allow packaging of Vpx into HIV-1 derived virus particles, we generated a chimeric HIV-1 vector harboring the p6 domain of the SIVsmmPBj virus. However, Vpx did not enhance transduction of monocytes with this chimeric HIV-1 vector, although efficient packaging was demonstrated [3], [11]. The block occurred after reverse transcription of vector RNA and before translocation of the PIC into the nucleus, similar to that previously described for HIV-1 wild-type vectors or for SIVsmmPBj vectors lacking Vpx [1], [3], [11], [19]. We considered that Vpx might not be present in the HIV-1 PIC to enable trafficking of the PIC into the nucleus, since Vpx requires interaction with other virus specific components such as integrase, MA or RNA. Therefore, two further strategies were pursued to assign functional activities of Vpx to HIV-1-derived vectors. First, we achieved packaging into HIV-1 vector particles by fusing PBj Vpx to HIV-1 Vpr, generating a HA-Vpr/Vpx fusion protein. Like Vpx, Vpr has been described to be present in the PIC and thus might transfer the Vpx protein into the HIV-1 PIC [12], [16]–[18]. Translocation of the PIC into the nucleus can directly be linked to the nuclear localization of Vpx and Vpr [34], [35], and this capacity was retained for the fusion protein HA-Vpr/Vpx. Furthermore, the Vpx part within the HA-Vpr/Vpx fusion protein was still functional in terms of enabling efficient transduction of monocytes, as proven in the context of the PBj4xko-EGFP vector. However, the fusion protein HA-Vpr/Vpx did not enhance transduction of monocytes or monocyte-derived cells if packaged into an HIV-1 vector. We concluded that beside Vpx, other viral components of the PBj-virus are required for efficient monocyte transduction.

Secondly, we used virus-like particles to assign Vpx functions to HIV-1-derived vectors, thus allowing monocyte transduction. Goujon *et al.* showed that SIVmac Vpx can be functionally provided *in trans* by pre-incubation of dendritic cells (DCs) with non-infectious SIVmac-derived virus-like particles (VLPs), which were simply used as carriers of the Vpx proteins [13], [14]. We investigated the suitability of SIVsmmPBj Vpx-containing VLPs derived from SIVsmmPBj to enhance transduction of monocytes by HIV-1 vectors. Remarkably, upon pre-incubation with Vpx-supplemented SIVsmmPBj-derived VLPs, the transduction efficiency of HIV-1 vectors was enhanced 10-fold, which is in line with a very recent publication [36]. By using increasing amounts of VLPs, we could show that the transduction efficiency was dose-dependent. Transduction with HIV-1 vectors was also mediated when the PBj-derived VLPs were supplemented with the HA-Vpr/Vpx fusion protein, but not by supplementation of HIV-1-derived VLPs with HA-Vpr/Vpx (data not shown). Thus, as already concluded from the transduction experiments using Vpx directly packaged into HIV-1 vector particles, it is evident that the Vpx protein is only capable of mediating monocyte transduction if it is delivered in the background of SIVsmmPBj capsids. Whether SIVsmmPBj capsid components are required for efficient release of Vpx from the particles to the cytoplasm, for a certain activation of Vpx, or for nuclear localization of the PIC, remains still elusive.

In contrast to our findings, Sharova *et al.* [37] observed an enhancing effect when Vpx was co-packaged into HIV-1 particles. In this case, no further viral components of SIVsmmPBj were necessary, indicating that the restriction mechanism described by Sharova *et al*. for macrophages may be different from the restriction block present in monocytes investigated by us. In our hands, packaging of Vpx into HIV-1 particles was only possible after insertion of the p6 domain into the HIV-1 genome [11] or after fusion of Vpx to Vpr, thus preventing direct comparison of the function of Vpx-containing HIV-1 particles.

Remarkably, PBj Vpx did also enable full replication of HIV-1 wild-type virus on human monocytes. Single pre-incubation of monocytes with PBj-derived Vpx-supplemented VLPs was sufficient to promote full replication of a CCR5-tropic HIV-1 virus comparable to that of SIVsmmPBj1.9 wild-type virus encoding Vpx. There are several possible explanations for this observation. It might be possible that the initial infection step during virus replication is most crucial and that once it has entered the nucleus the virus may efficiently replicate in the infected cell. Another explanation might be that Vpx has a sustained effect in the target cells, allowing several rounds of infection. This hypothesis is supported by our own observations that Vpx containing VLPs, delivered to monocytes 20 h before the HIV-1-derived vector still enabled efficient single round transduction (data not shown).

Possible modes of action of Vpx could be by counteracting the function of cellular antiviral genes, or induction of important cofactors lacking in monocytes and required e.g. for cell cycle progression [38], [39]. However, surface marker expression indicative for induction of differentiation or activation like CD11c, CD14, CD86, DC-SIGN or MHC-II were not or only marginally influenced by Vpx provided by VLPs, especially at day two after isolation when all of the cells are still monocytes. Similar results have been described for dendritic cells treated with Vpx [13]. Therefore, we suggest that counteraction of cellular antiviral genes, rather than induction of differentiation or activation, constitutes the functionality of Vpx.

Several proteins mediating retroviral post-entry and post-integration blocks of HIV-1 in monocytes or DCs have been described. As the suppression of protein expression by specific siRNAs against different members of the APOBEC family, namely A3G, A3A and A3F, restored the susceptibility of monocytes and DCs to HIV-1 infection [6], [10], [40], we analyzed the influence of Vpx on APOBEC expression. Recent experiments indicated that pre-incubation of monocytes with Vpx-supplemented PBj-VLPs did not alter either mRNA levels of A3G, A3A, A3F, A3C or Trim5α_hu_, protein levels of A3A or A3G, although the transduction efficiency of HIV-1 was significantly enhanced (data not shown). From these experiments we concluded that Vpx may not act as a direct antagonist of APOBEC proteins. APOBEC3G has been described to impair the initiation of HIV-1 reverse transcription and/or processivity of reverse transcription [41]. The accumulation of RT products in DCs was strongly enhanced in the presence of Vpx [13], [14], [42], [43], which indicates that Vpx counteracts the restriction during reverse transcriptions. Since transduction of monocytes was also enhanced when the Vpx containing VLPs were added up to 20 h post HIV-1 transduction (data not shown), it might be likely that the restriction occurs just before translocation of the PIC in the nucleus or even in the nucleus, before integration. However, it remains still elusive in which way Vpx might counteract different APOBEC or Trim5α restriction pathways, considering that we have not observed a change in APOBEC gene expression.

For non-human primate cells, additional species specific pre- and post- integration blocks of retroviruses, such as Trim5α have been described [29], [44]–[46]. To analyze, whether Vpx does circumvent restriction of HIV-1 in non-human primate cells, we isolated CD14^+^ cells from rhesus macaques or Pig-tailed macaques. In Pig-tailed macaque cells, HIV-1 infection is not restricted by Trim5α as in rhesus macaque cells [31]. Remarkably, we could show an enhanced transduction of monocytes from both simian species using HIV-1-derived vectors. Pre-incubation with VLPs not supplemented with Vpx did not increase HIV-1 transduction efficiency. Thus a simple saturation of restriction factors by Gag or Pol proteins such as described for simian Trim5α [47], [48] can be excluded. Vpx allowed a single-round infection of non-human primate monocytes but did not enable full replication of HIV-1 wild-type virus in monocytes from the simian species tested. We conclude that the Vpx protein counteracts restriction of early steps in HIV-1 infection, such as entry, reverse transcription [14] or nuclear import [35], [49], in human as well in non-human primate monocytes. In human monocytes, this is sufficient for complete virus replication. However, HIV-1 replication is not enabled in non-human primate monocytes, which may be attributed to other post-integration blocks of HIV-1 replication which are not counteracted by Vpx, such as simian APOBEC proteins [44], [50].

We suggest that the function of Vpx is to antagonize an antiviral restriction factor present in primary human monocytes. Single-round and productive infection by HIV-1 can be attained by providing the PBj Vpx protein *in trans* using PBj-derived but not HIV-1-derived particles, the molecular basis for this specificity remains unknown. Using Vpx-supplemented PBj-derived VLPs, the early restriction block of HIV-1 is also circumvented in non-human primate monocytes. However, additional post-integrations blocks not present in human monocytes restrict full virus replication.

## Materials and Methods

### Plasmids

The env-deficient SIVsmmPBj1.9 vector constructs pPBjΔE-EGFP and pPBj4xko-EGFP, as well as the expression constructs pcHA-Vpx and pcwt-Vpx were described previously [3], [11]. The HIV-1-derived transfer vector plasmid pHR-CMV-EGFP [51] and the respective packaging construct pCMVdR8.9 [52] were kindly provided by U. Blömer. The SIVsmmPBj1.9-derived packaging construct PBj-psi10 is based on the *env*-deleted molecular clone pPBjΔenv [3]. For construction of PBj-psi10, a CMV promoter was used to replace the 5′ LTR, and the 3′ LTR was replaced with the bovine growth hormone polyadenylation site using *BsaB I* and *Not I* restriction sites. The accessory genes *vif*, *vpx*, *vpr* and *nef* were deleted by applying fusion PCR, while the regulatory genes *tat* and *rev* were maintained. Furthermore, 155 bp between the CMV promoter and the major splice donor (SD) site as well as 59 bp between SD and *gag* were deleted by fusion PCR, preserving exclusively the primer binding site and the SD. Primer sequences are available upon request.

The HA-Vpr/Vpx-expression construct was compiled by fusing PCR fragments generated from HIV-1-NL_4-3_ and SIVsmmPBj1.9 DNA using 5′ CGC GGATCC GCC ACC ATG TAC CCC TAC GAC GTG CCC GAC TAC GCC GGC GAA CAA GCC CCA GAA GAC 3′ (forward) and 5′ CCT GGG ATC TGA CAT GCC GCC GGA TCT ACT GGC TCC ATT TCT3′-3′ (reverse) primers to amplify the Vpr encoding region Vpr and 5′ AAT GGA GCC AGT AGA TCC GGC GGC ATG TCA GAT CCC AGG GAG A 3′ (forward) and 5′ CCG CTC GAG TTA TGC TAG TCC TGG AGG GGG AGG AG 3′ (reverse) to amplify the Vpx encoding region, respectively. For fusion of the obtained Vpr and Vpx specific PCR products, the Vpr specific forward primer and the Vpx-specific reverse primer were applied. The amplification product spanning the respective HA-Vpr/Vpx encoding gene was inserted into the mammalian expression vector pcDNA3.1(+) (Invitrogen, Carlsbad, CA, USA) using the restriction sites *BamH I* and *Xho I*. All expression constructs were confirmed by DNA sequence analysis (MWG Biotech, Ebersberg, Germany).

### Cell lines

293T (ATCC; SD 3515), HT1080 (ATCC; CCL121) and HeLa (ATCC CCL-2) were maintained in Dulbecco's modified Eagle's medium (DMEM) supplemented with penicillin (100 U/ml), streptomycin (100 µg/ml), L-glutamine (2 mM) and 10% FCS.

### Generation and titration of vector and virus-like particles

For generation of SIVsmmPBj-derived vectors, 293T cells were co-transfected with one of the vector-encoding plasmids described above and pMD.G [53] coding for the vesicular stomatitis virus glycoprotein G (VSV-G) by the calcium phosphate transfection method. HIV-1-derived vectors were generated likewise but using the vector encoding plasmid pHR-CMV-EGFP and the packaging construct pCMVΔR8.9. For production of PBj- or HIV-1-derived VLPs, the respective packaging construct was co-transfected with pMD.G. For supplementation of SIVsmmPBj1.9 or HIV-1 vectors or VLPs with Vpx, HA-Vpr or HA-Vpr/Vpx, the respective expression plasmid was additionally co-transfected into vector producing cells. After 48 hours, the vector containing supernatants were collected, filtrated (0.45 µm filter, Sartorius, Göttingen, Germany), concentrated by ultracentrifugation (125,000 x g, 2 h, 4°C) through a 20% sucrose cushion and subsequently stored at −80°C. For titration, vectors were added in serial dilutions to permissive HT1080 cells seeded in 24-well plates. After 4 h, culture medium was exchanged. Four days later, cells were monitored for EGFP expression by FACS analysis. The amount of VLPs was determined by Lenti RT Activity kit (Cavidi AB, Uppsala, Sweden) and normalized in comparison to PBj- or HIV-1-vectors of known infectivity. For incubation experiments, the amount of VLPs per cell was given as MOI-equivalents (MOIeq).

### Isolation, transduction and infection of primary human and simian monocytes

Primary human CD14^+^ monocytes were isolated from purified PBMC from healthy donors by negative depletion, and monocytes of rhesus macaques or Pig-tailed macaques by positive selection employing magnetic activated cell sorting (MACS) using the Monocyte Isolation Kit II or the isolation Kit for non-human primate cells, respectively (Miltenyi Biotec, Bergisch Gladbach, Germany). Housing of monkeys and blood donation was maintained in accordance with the German animal license regulations (Tierschutzgesetz). Purity of isolated monocytes was assessed by flow cytometry (FACScan™, Becton Dickinson) analysing expression of the monocyte marker CD14 using a specific phycoerythrin-conjugated monoclonal antibody (BD Bioscience, Heidelberg, Germany). Monocytes were seeded at a density of 4×10^5^ cells/well into 48-well tissue culture plates (BD Falcon, Bedford, MA, USA) and maintained in VLE-RPMI1640 (Biochrom AG, Berlin, Germany) supplemented with penicillin (100 U/ml), streptomycin (100 µg/ml), L-glutamine (2 mM), non essential amino acids (Gibco), 10 ml/L OPI (oxaloacetic acid,. pyruvate and insulin; SIGMA-Aldrich, Taufkirchen, Germany) and 10% human-AB serum (Biochrom AG, Berlin, Germany). For cultivation of simian cells, culture medium was supplied with 10% autologous serum. In some experiments, at the desired day after isolation, monocytes or monocyte-derived cells were pre-exposed for two hours to VLPs where the amount of VLPs per cell was given as MOI-equivalents (MOIeq). For transduction, cells were exposed for 4 h to SIVsmmPBj1.9- or HIV-1-derived vectors. EGFP expression of vector-transduced cells was measured by FACS analysis 6 days post transduction. For analysis of virus replication, monocytes were infected in triplicate on day 1 post isolation with an MOI of 0.5 with HIV-1 or SIVsmmPBj strains and cultured up to day 21. Before infection with the respective wild type viruses, non-human primate cells were pre-stimulated for 8 h with 20 ng/ml TPA. To determine the amount of SIVsmmPBj and HIV-1, supernatants (200 µl) were collected and the amount of reverse transcriptase activity was determined by Lenti RT Activity kit (Cavidi AB, Uppsala, Sweden).

For determination of cell surface marker expression, primary monocytes were isolated as described above and seeded at a density of 3×10^6^ cells/well into 6-well tissue culture plates (BD Falcon, Bedford, MA, USA). On day 2, 5 and 9 post isolation, cells were cultivated for 48 h in the presence of PBj-derived VLPs at an MOIeq of 1. The cells were stained with αCD11c-FITC (Acris GmbH, Herford, Germany) αCD14-PE (DAKO, Hamburg, Germany), αCD86-PE (BD-Pharmingen, Heidelberg, Germany), αCD209-FITC (Milteny Biotec, Bergisch Gladbach, Germany) or αMHC-II-FITC (DAKO) and analyzed by flow cytometry (LSR II; Becton Dickinson, Heidelberg, Germany). During data analysis with FCS-Express (DeNovo Software, Los Angeles, USA) the isotype controls were matched.

### Detection of virion proteins by Western blotting

Vector or VLP-containing supernatants were clarified by filtration (0.45 µm filter) and concentrated by ultracentrifugation through a 20% sucrose cushion (125,000 x g, 2 h, 4°C). Subsequently, viral particles were resuspended in 1 ml of PBS (4°C) and ultracentrifuged through 12 ml of a 20% sucrose cushion (140,000 x g, 1 h, 4°C).

For Western blot analysis, vector particles were resuspended in ice-cold lysis buffer (50 mM HEPES [pH 7.4], 125 mM NaCl, 0.1 mM phenylmethylsulphonyl fluoride, 0.2% NP40), and transfected 293T cells were lysed in ice-cold RIPA buffer (Tris 50 mM, NaCl 150 mM, SDS 0.1%, Na-Deoxycholate 0.5%, 0.1 mM phenylmethylsulphonyl fluoride, NP40 1%), both supplemented with protease inhibitor cocktail (Roche, Mannheim, Germany). Cell lysates were additionally sonicated and protein concentrations were determined by the Bradford method.

Samples were denatured (95°C, 5 min) and separated on NuPAGE Novex 4–12% SDS-polyacrylamide gels (Invitrogen, Carlsbad, CA, USA). Following electrophoresis, proteins were transferred to Hybond ECL nitrocellulose membrane (GE Healthcare, München, Germany) by electroblotting, incubated in blocking buffer (5% nonfat dry milk in PBS, 0.1% Tween 20) at room temperature for 1 h and then over night at 4°C with the appropriate primary antibody. Anti-HIV-2-Vpx monoclonal antibody (NIH AIDS Research and Reference Reagent Program, Rockville, USA) was used 1∶50, anti-HA monoclonal antibody (Covance, Princeton, NJ, USA) was diluted 1∶5,000 and anti VSV-G (SIGMA-Aldrich, Taufkirchen, Germany) was diluted 1∶10,000. Protein-bound antibodies were detected with HRP-conjugated specific secondary antibodies (diluted 1∶7,500 respectively), followed by enhanced chemiluminescence analysis (GE Healthcare, München, Germany).

## Supporting Information

Figure S1 HA-tagged Vpr, Vpx and the Vpr/Vpx fusion protein are localized in the nucleus. HeLa cells transfected using FuGENE reagent with the expression constructs indicated on the left. Two days after transfection, cells were fixed in 4% paraformaldehyde, permeabilized in 0.1% Triton-X-100, and blocked with Image-iT. The HA-tagged Vpx, Vpr or Vpr/Vpx fusion protein were stained with an anti-HA antibody. Monoclonal secondary antibody anti-mouse Alexa Fluor 488, was used together with a Rhodamine Phalloidine solution. Subsequently, 4,6-diamidino-2-phenylindole staining was performed. Finally, cells were embedded in Mowiol and analyzed by confocal laser scanning microscopy. (α-HA) Indirect immunofluorescence using anti-HA antibodies and Alexa Fluor 488 conjugated secondary antibody. (DAPI) Staining of nuclei by 4, 6-diamidino-2-phenylindole. (Rhodamin) Staining of cytoplasmatic microfilaments by rhodamin-phalloidine.
(3.09 MB TIF)
