# Supplementary figures and images for: Restriction of HIV-1 Replication in Monocytes Is Abolished by Vpx of SIVsmmPBj
Source: PLoS One. 2009 Sep 21;4(9):e7098. doi: 10.1371/journal.pone.0007098 (PMC2741571; doi:10.1371/journal.pone.0007098)

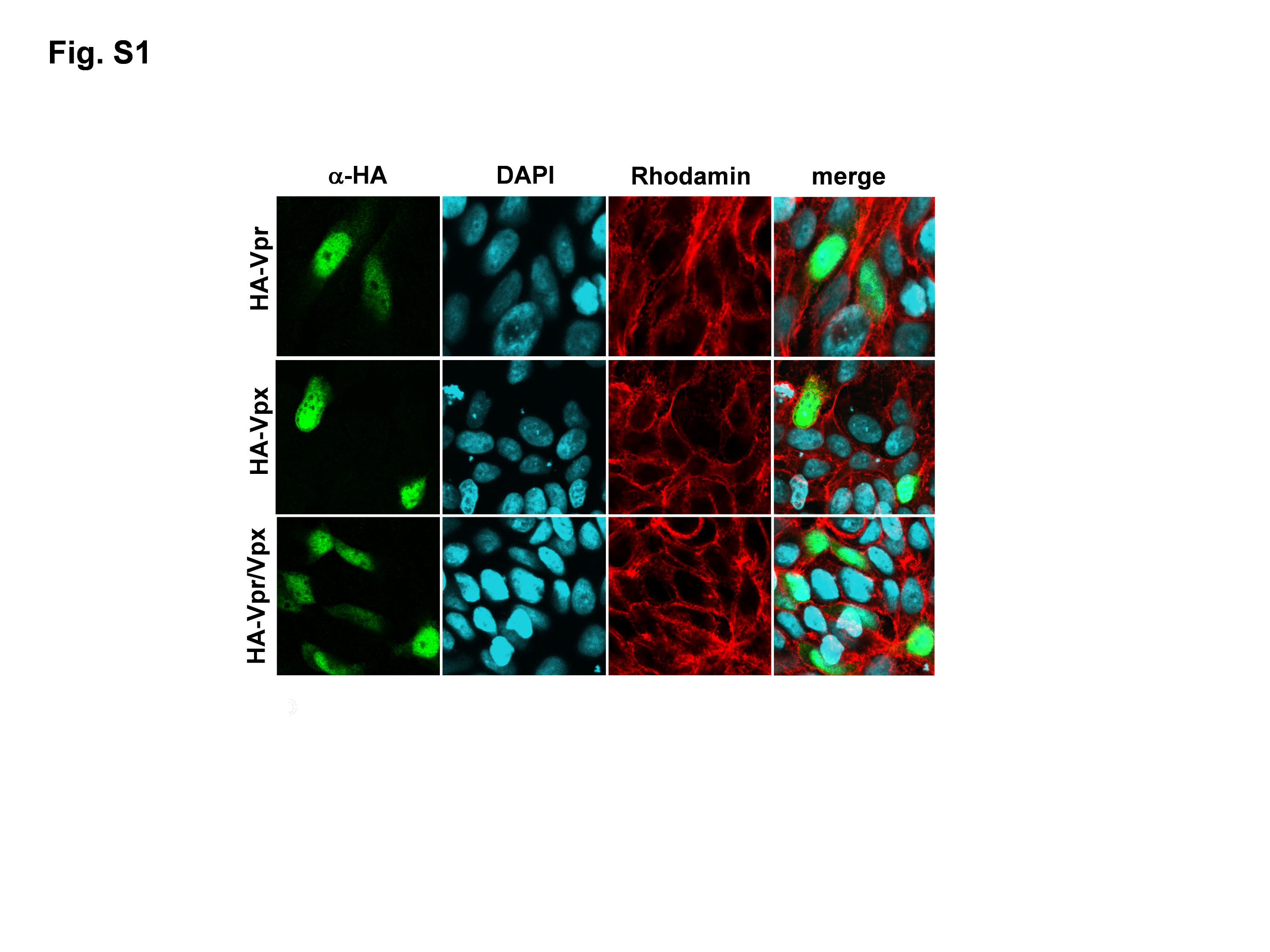

Supplement: Figure S1 — HA-tagged Vpr, Vpx and the Vpr/Vpx fusion protein are localized in the nucleus. HeLa cells transfected using FuGENE reagent with the expression constructs indicated on the left. Two days after transfection, cells were fixed in 4% paraformaldehyde, permeabilized in 0.1% Triton-X-100, and blocked with Image-iT. The HA-tagged Vpx, Vpr or Vpr/Vpx fusion protein were stained with an anti-HA antibody. Monoclonal secondary antibody anti-mouse Alexa Fluor 488, was used together with a Rhodamine Phalloidine solution. Subsequently, 4,6-diamidino-2-phenylindole staining was performed. Finally, cells were embedded in Mowiol and analyzed by confocal laser scanning microscopy. (α-HA) Indirect immunofluorescence using anti-HA antibodies and Alexa Fluor 488 conjugated secondary antibody. (DAPI) Staining of nuclei by 4, 6-diamidino-2-phenylindole. (Rhodamin) Staining of cytoplasmatic microfilaments by rhodamin-phalloidine. (3.09 MB TIF) [file pone.0007098.s001.tif]
